# RRBM-YOLO: Research on Efficient and Lightweight Convolutional Neural Networks for Underground Coal Gangue Identification

**DOI:** 10.3390/s24216943

**Published:** 2024-10-29

**Authors:** Yutong Wang, Ziming Kou, Cong Han, Yuchen Qin

**Affiliations:** 1 School of Mechanical Engineering, Taiyuan University of Technology, Taiyuan 030024, China; wyt_0028@163.com (Y.W.); chenerlover@163.com (Y.Q.); 2 Shanxi Provincial Engineering Laboratory for Mine Fluid Control, Taiyuan 030024, China

**Keywords:** coal gangue recognition, YOLOv8, lightweight module RepGhost, attention mechanism MCA, low light enhancement

## Abstract

Coal gangue identification is the primary step in coal flow initial screening, which mainly faces problems such as low identification efficiency, complex algorithms, and high hardware requirements. In response to the above, this article proposes a new “hardware friendly” coal gangue image recognition algorithm, RRBM-YOLO, which is combined with dark light enhancement. Specifically, coal gangue image samples were customized in two scenarios: normal lighting and simulated underground lighting with poor lighting conditions. The images were preprocessed using the dim light enhancement algorithm Retinexformer, with YOLOv8 as the backbone network. The lightweight module RepGhost, the repeated weighted bi-directional feature extraction module BiFPN, and the multi-dimensional attention mechanism MCA were integrated, and different datasets were replaced to enhance the adaptability of the model and improve its generalization ability. The findings from the experiment indicate that the precision of the proposed model is as high as 0.988, the mAP@0.5(%) value and mAP@0.5:0.95(%) values increased by 10.49% and 36.62% compared to the original YOLOv8 model, and the inference speed reached 8.1GFLOPS. This indicates that RRBM-YOLO can attain an optimal equilibrium between detection precision and inference velocity, with excellent accuracy, robustness, and industrial application potential.

## 1. Introduction

Coal gangue is a byproduct generated in the process of coal extraction, accounting for about 15% of the original coal production. Compared to ordinary coal, it has low carbon content, low calorific value, and a hard texture [1,2,3]. The massive storage of coal gangue not only wastes land resources but also causes situations such as spontaneous combustion, rainfall, and mudification, causing significant damage to the environment [4]. Hence, the effective identification and full utilization of coal gangue represent a crucial challenge for the coal sector and are key areas of focus for sustainable research.

Conventional approaches for coal gangue identification encompass manual inspection, image recognition, and ray-based recognition techniques. However, the manual selection of waste materials leads to high labor intensity and low efficiency among workers, and the leakage of waste materials often occurs. Moreover, while X-ray and gamma ray detection methods can attain greater precision, these methods are subject to ionizing radiation, which can cause significant physical damage to operators. Therefore, their application is somewhat limited.

In recent years, advancements in image processing technology and machine learning have led to extensive research on coal gangue recognition methods utilizing images and machine learning algorithms. Traditional machine learning approaches for coal gangue recognition primarily employ techniques such as K-means clustering, support vector machines, random forests, etc., utilizing grayscale images or texture features for training [5]. Li et al. [6] proposed a method for preprocessing low-quality coal gangue pictures based on joint improvement calculation. K-means clustering division is used to compare the division results of different preprocessing strategies utilizing data entropy and auxiliary closeness measures in arrangements to play down noise, enhance subtle picture elements, and accomplish great division. Zhao et al. [7] proposed a coal gangue recognition strategy based on a combination of X-ray transmission and diffraction standards. Distinctive identified highlights were extricated for distinctive molecule sizes, and a PSO-SVM demonstration was set up for distinguishing coal and gangue. Lal et al. [8] planned a two-dimensional autoencoder (2D-AE) for ghostly information dimensionality and utilized the irregular timberland strategy for coal gangue identification. The identification impacts of CART, KNN, SVM, and AdaBoost were compared. These tests demonstrated that the combination of 2D-AE and RF has the most elevated normal exactness.

In spite of the fact that numerous advancements have been made, coal gangue identification still faces issues such as troublesome highlight extraction and parameter alteration, low detection accuracy, moderate handling speed, overfitting of models, and weak generalization capacity [9]. With noteworthy advances in profound learning hypotheses, this approach has steadily become the standard calculation method within the field of coal gangue distinction [10]. The current coal gangue recognition task mainly faces two challenges: The first challenge comes from the large amount of coal produced underground and the harsh environment. The existing machine vision coal gangue identification calculation features a small open field, low highlight extraction capacity, a moderate preparation of the convergence speed, and a small test estimate, resulting in poor generalization ability, a low gangue recognition rate, and a high mixing rate. The second challenge comes from the time-consuming algorithmic complexity and high hardware requirements in the recognition process, which place high demands on the system’s execution speed.

The SSNet-CG proposed by Wang et al. [11] can rapidly capture and intertwine the characteristics of coal gangue to realize the exact division and quick identification of coal gangue attachment boundaries. Wang et al. [12] introduced a data-oriented network for coal gangue detection called SSD-BSP. Lai et al. [13] proposed and made strides in improving a coal gangue division strategy combining Mask R-CNN and multispectral imaging, but the recognition exactness and proficiency of the spine arrangement must be studied further.

Zhang et al. [14] studied the input picture utilizing three information enlargement strategies: mosaic constriction vector information increase, cosine tempering, and name smoothing. Yang [15] and others built a real-time detection model of the average coal gangue within the complex environment of a coal mine by improving upon YOLOv5. Zeng et al. [16] proposed a Laplacian image enhancement algorithm to sharpen contours and enhance feature extraction, but they did not take into account the harsh lighting environment underground, making it inconceivable to repair complex debasement designs such as clamor, artifacts, and color twisting covered up within the dull environment or presented due to brightness upgrades.

Xue et al. [17] utilized ResNet18’s lightweight organization as the spine, including the extraction organization of YOLOv3, and proposed a ResNet18-YOLO coal gangue location calculation. Qiang et al. [18] examined a coal gangue detection strategy based on YOLOv4, which made strides regarding the bounding box and sifting and included pyramid layers. Peng et al. [19] considered a clever classification strategy of coal gangue based on multispectral imaging innovation and progressed the YOLOv5 (with an included seSE attention instrument module) question location. Gui et al. [20] proposed a real-time securing framework for coal gangue picture recognition based on YOLOv5 calculation (consolidating a CBAM attention instrument). However, simplifying the network backbone may potentially compromise detection accuracy, as sampling operations can readily result in the loss of essential features. Furthermore, the fusion attention mechanism either necessitates the incorporation of additional parameters or neglects the modeling of both channel and spatial dimensions. Consequently, the aforementioned algorithms face challenges in achieving a harmonious balance among network lightweighting, feature extraction, and model convergence performance.

In response to the aforementioned scenario, a high-efficiency and lightweight convolutional neural network, named RRBM-YOLO, is proposed for the purpose of underground coal gangue identification. The primary contributions of this paper are as follows:

■ At present, there is no publicly available coal gangue dataset. Custom coal gangue image samples were created in two scenarios: ordinary lighting and simulated underground lighting with poor lighting conditions. Images were preprocessed using methods such as screening, sample expansion, and Retinexformer, a dark light enhancement algorithm combined with retinal theory. Based on this, images were annotated to construct a coal gangue dataset, promoting knowledge exchange, technological innovation, and application development, providing better solutions for coal gangue management and environmental protection.
■ A “hardware friendly” coal gangue image recognition algorithm was proposed, which uses YOLOv8 as the backbone network and integrates lightweight module RepGhost, the repeated weighted bi-directional feature extraction module BiFPN, and the multi-dimensional attention mechanism module MCA. This is a topic that has received limited attention in the literature but urgently needs to be solved in practical working conditions.
■ Coal gangue may change in color, surface, and other viewpoints across numerous places. To progress the generalization capacity of the proposed algorithm, different types of datasets were supplanted to upgrade the versatility of the model. Great detection results were obtained that show that the proposed improved model also has great industrial application potential in other research areas.

Within the following chapters, Section 2 presents the general system and advancement points of interest of the calculation. Section 3 presents the information planning and gives a point-by-point clarification of the test setup. Section 4 analyzes the test results to illustrate the adequacy of the proposed strategy. At long last, Section 5 summarizes the research discoveries and considers future work.

## 2. The Proposed Methods

### 2.1. Improved YOLOv8 Model

**Figure 1:**
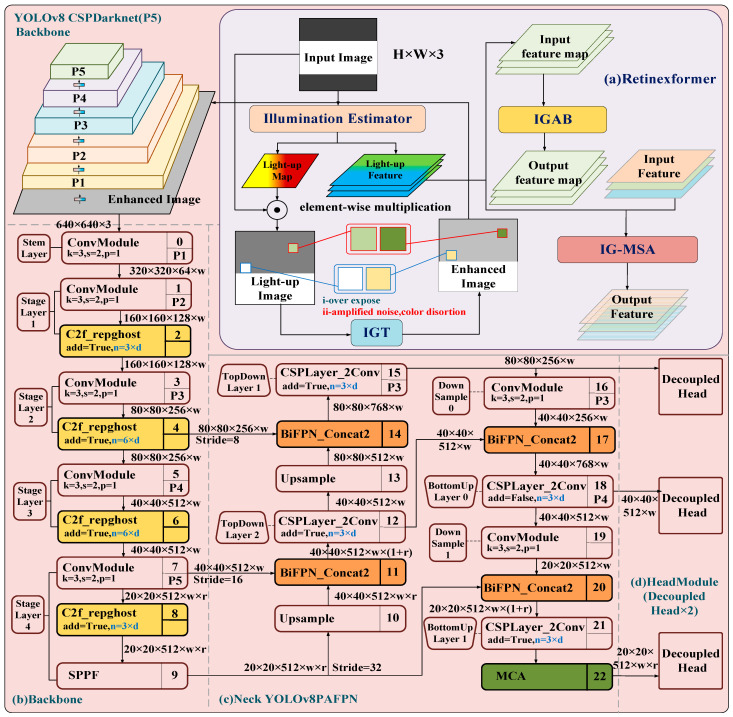
General graph of the improved YOLOv8 algorithm structure proposed.

**Figure 2:**
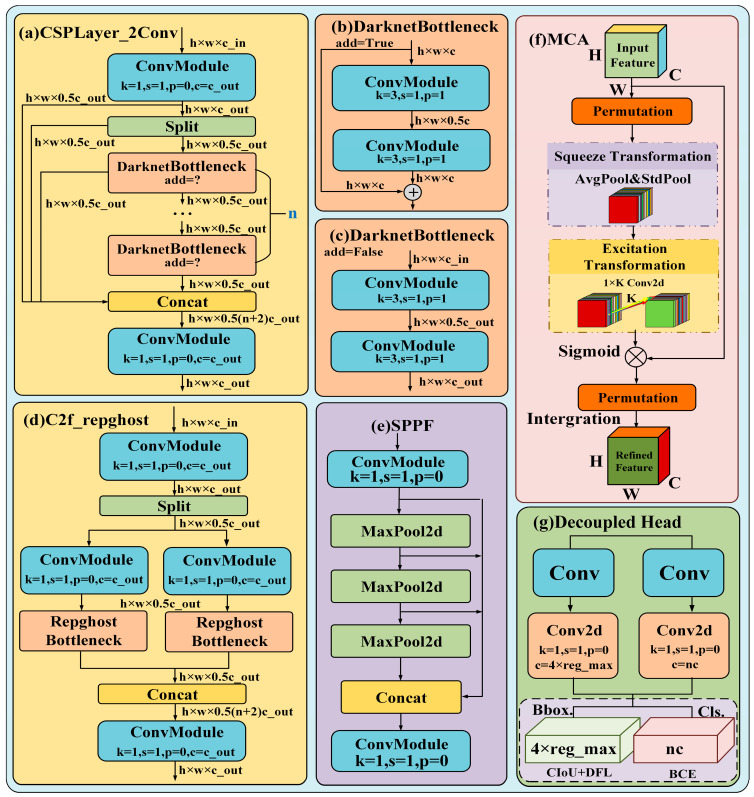
Proposed improved YOLOv8 network structure diagram. (**a**) Convolution module, used to improve network efficiency and performance. (**b**) The output feature maps of the two convolution modules in DarknetBottleneck are merged element by element to preserve more feature information. (**c**) The output feature maps of the two convolution modules in DarknetBottleneck use more complex merging strategies, such as Concatenation or other methods, to optimize gradient propagation and model performance. (**d**) Refactoring the C2f module in Backbone to C2f_repghost to achieve an efficient lightweight CNN. (**e**) The spatial pyramid fast pooling module is used for multi-scale feature extraction. (**f**) Simultaneously promote attention in the dimensions of channel, height, and width. (**g**) Decoupling head structure, separating classification and detection heads.

This study made the following improvements to the original YOLOv8: considering the harsh underground lighting environment, the input was first preprocessed by the dim light enhancement algorithm Retinexformer, and then the improved YOLOv8 model was used for object detection (Figure 1). Specifically, for the improvement part, it is necessary to refactor the C2f module in Backbone into C2frepghost (Figure 2d) to achieve an efficient and lightweight CNN; define the class of BiEPN Concat2 to replace Concat in the original YOLOv8Neck, concatenating bi-directional cross-scale associations and quick normalization combinations to attain straightforward and quick multi-scale input combinations (Figure 1c); and include an MCA layer after the final layer of C2f, which advancing attention in the channel, height, and width dimensions (Figure 2f). The above improvements make the model more accurate and faster to execute. The schematic graph of the proposed model is shown in Figure 1.

YOLOv8 is the latest research achievement of the current One Stage YOLO series object detection algorithms, which has made strides on the premise of YOLOv5’s calculation [21]. The spine organizer and neck have replaced YOLOv5’s C3 structure with a C2f structure with richer gradient flow (Figure 2a–c). The head has changed from the initial coupling head to the current standard decoupling head structure, isolating the classification and detection heads (Figure 2h), and the original SPP has been replaced with SPP Fast (Figure 2e). These improvements result in a higher mAP and faster inference speed on the COCO dataset.

### 2.2. Dim Light Image Enhancement Module Retinexformer

**Figure 3:**
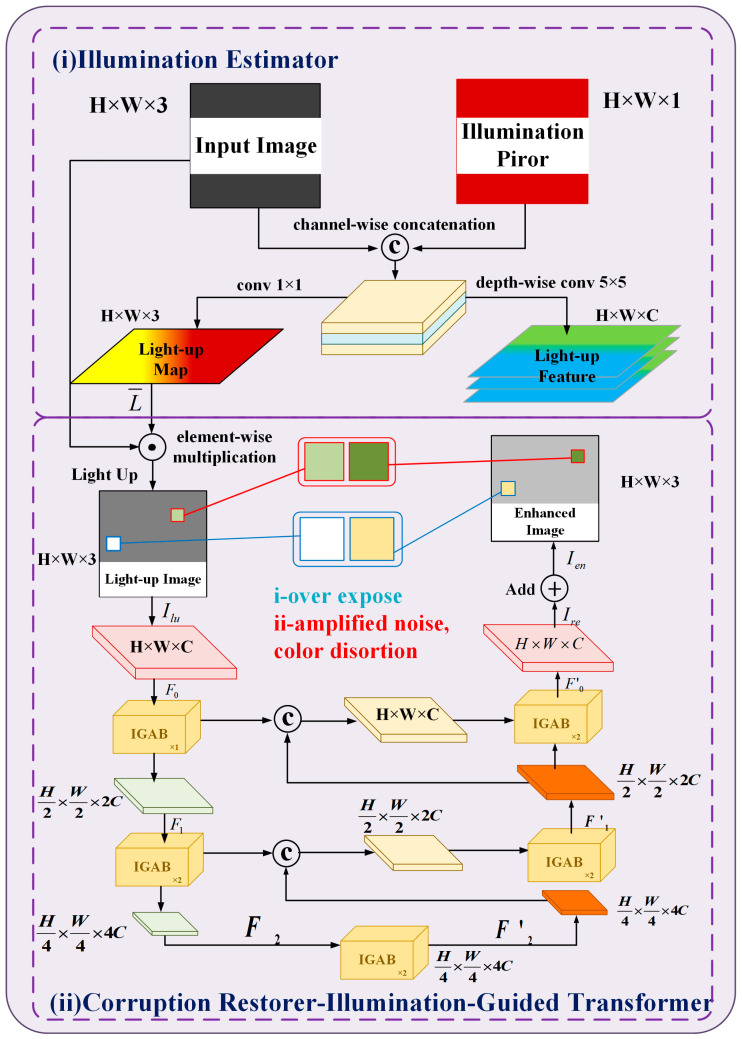
Method overview. The Retinexformer adopts a proposed ORF consisting of a light estimator (**i**) and a damage recovery device (IGT) (**ii**).

This article adopts a new method for enhancing low-light images—Retinexformer. This method first developed a single-stage framework (ORF) based on retinoic acid. ORF (which can estimate lighting information and illuminate low-light images) employs a harm restorer to reduce clamor, artifacts, underexposure or overexposure, and color twisting. Then, an illumination-guided transformer (IGT) is used to simulate remote dependency relationships. Finally, the IGT is inserted into the ORF as a damage restorer to derive a retexer [22]. The calculation guideline is shown in Figure 3.

**Figure 4:**
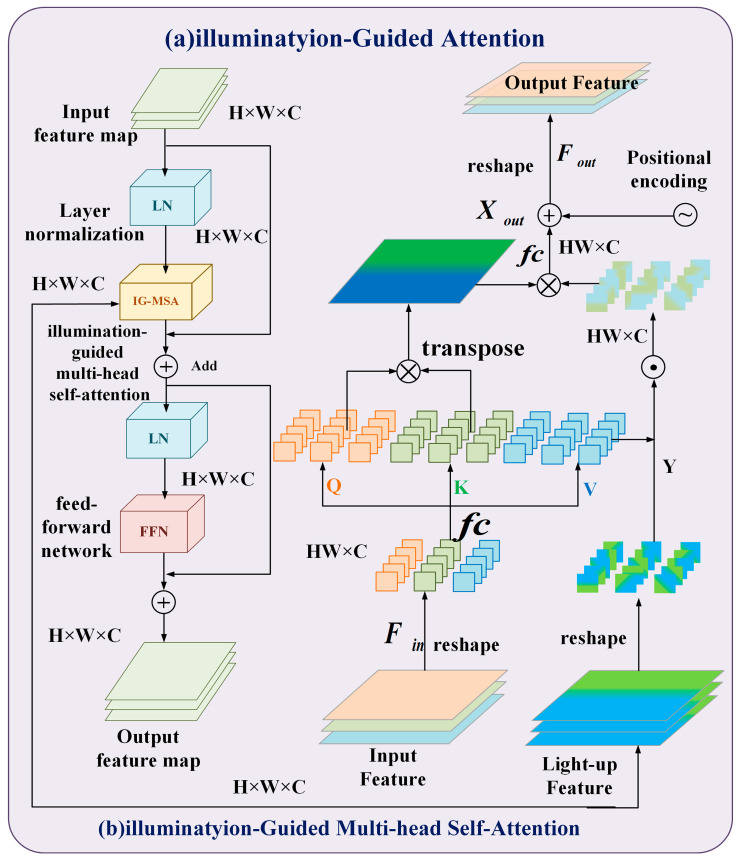
Network structure details. (**a**) The basic unit of IGT, IGAB, consists of two layers of normalization (LN), IG-MSA and feedforward network (FFN). (**b**) IG-MSA uses lighting representations captured by ORF to guide the calculation of self-attention.

It is well known that ORF is prepared end-to-end in a one-stage way, redressing the first Retinex model by presenting annoyance terms in reflectivity and illuminance to mimic erosion. The key component of IGT is illumination-guided multi-head self-attention (IG-MSA) (Figure 4a). IG-MSA utilizes light representation to direct self-attention calculation and enhance the interaction between regions with different lighting levels (Figure 4b).

### 2.3. Lightweight Module RepGhost

The RepGhost module generates and fuses various feature maps through structural reparameterization techniques. The information fusion process is implicitly executed by addition operators, rather than leaving it to other convolutional layers, achieving implicit feature reuse and saving a lot of inference time.

**Figure 5:**
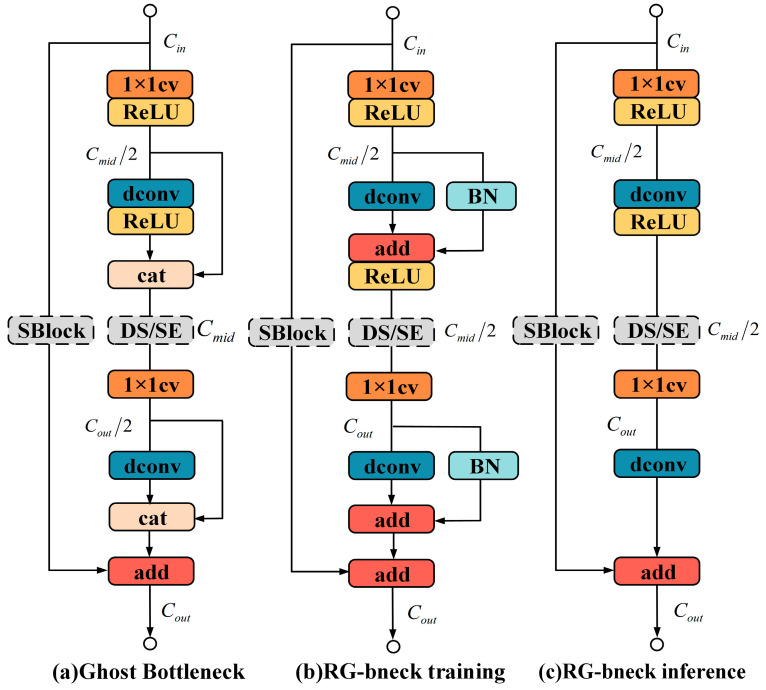
SBlock: skip connection block; DS: downsampling layer; SE: downsampling block; RG-bneck: RepGhost bottleneck. C_in_, C_mid_, and C_out_ represent the input, intermediate, and output channels of the bottleneck, respectively.

In terms of details, the RepGhost bottleneck (Figure 5b) maintains the same input and output channel numbers as the Ghost bottleneck (Figure 5a) but applies downsampling and SE on feature maps with reduced intermediate channels and deep convolution on feature maps with increased channels, expanding network capacity and making the RepGhost bottleneck more effective. Moreover, due to structural reparameterization, the RepGhost bottleneck only includes one shortcut and two branches of an operator chain (1 × 1 convolution, depth convolution, and ReLU) during the inference process (Figure 5c), resulting in lower memory cost and faster inference speed. In addition to the input and output layers, RepGhostNet stacks up the bottlenecks of RepGhost. A dense convolutional layer with 16 channels processes input data, and normal 1 × 1 convolution and average pooling stacks predict the final output [23].

### 2.4. Repeated Weighted Bi-Directional Feature Pyramid Network (BiFPN)

**Figure 6:**
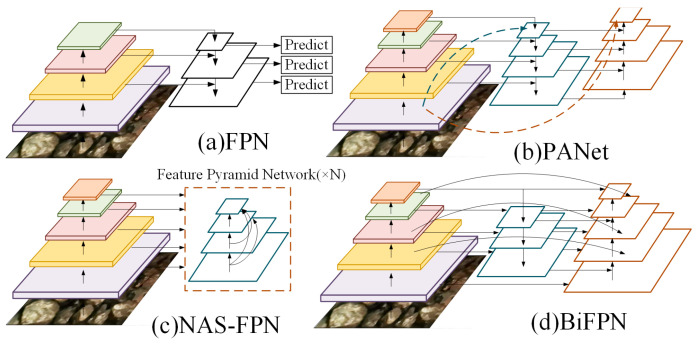
Feature network: (**a**) FPN [25] introduces a top-down path to fuse multi-scale features; (**b**) PANet [26] added an additional bottom-up pathway above FPN; (**c**) NAS-FPN [27] uses neural structure searching to find irregular feature network topologies and repeatedly applies the same block; (**d**) BiFPN, repeated weighted bi-directional features.

Multi scale feature fusion aims to aggregate features of different resolutions. The conventional FPN model transmits high-level solid semantic highlights in a top-down way to total multi-scale highlights (Figure 6a), but it is inherently restricted by the unidirectional data stream and requires precision. PANet includes an extra bottom-up way accumulation network to transmit low-level solid localization highlights, empowering the anticipated input outline to have both large semantic and positional data (Figure 6b). NAS-FPN employs neural engineering to seek for way better cross-scale highlight network topologies, but the look handle requires thousands of GPU hours, is time-consuming and labor-intensive, and the found network is unpredictable and troublesome to decipher or adjust (Figure 6c). In order to improve productivity and precision, the BiFPN evacuates hubs as if it were one input edge within the combination of distinctive highlight systems; an extra edge is included between the initial input and yield hubs at the same level to combine more highlights without adding too much cost. Consider each bi-directional (top-down and bottom-up) path as a highlight network layer and rehash the same layer various times to attain higher-level input combinations. Through the above method, bi-directional cross-scale associations and quick normalization combination capacities are associated, accomplishing basic and quick multi-scale highlight combination (Figure 6d) [24].

### 2.5. Multidimensional Collaborative Attention Module (MCA)

**Figure 7:**
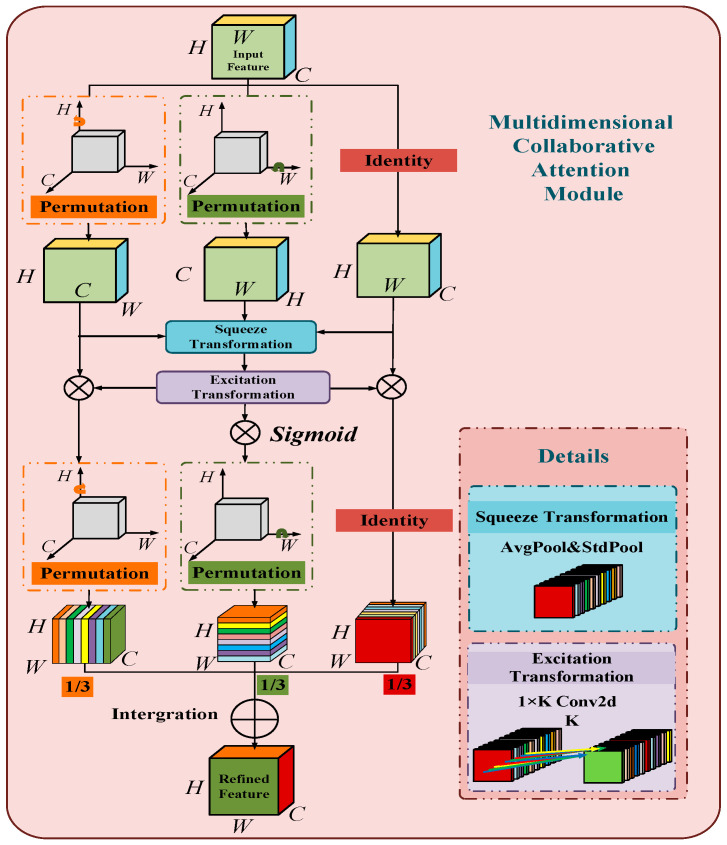
Schematic diagram of MCA module with three branches.

MCA is a novel, lightweight, and proficient multi-dimensional collaborative attention module that utilizes three-department engineering to advance attention in channel, stature, and width measurements simultaneously with nearly no extra overhead. It improved the problem of feature loss and improves the convergence performance of the model [28]. The top and middle branches capture the interactions between features in numerous spatial measurements (W and H), separately, and capture the distance conditions between the channel measurement and any spatial measurement through change. The foot branch is capable of capturing the interaction between channels. Finally, the yields of the three branches are basically the middle value of coordinates for accumulation, as shown in Figure 7.

**Figure 8:**
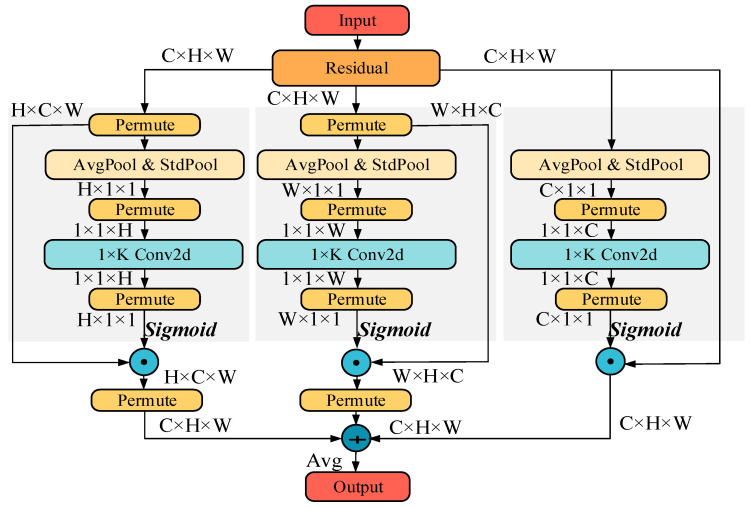
Diagram of proposed multi-dimensional collaborative attention (MCA) module.

The network has created a versatile combination component to blend the double cross-dimensional highlight reactions in squeeze transformations, upgrading the data substance and distinguishability of highlight descriptors. In addition, a gating instrument has been planned within the excitation transformation to adaptively decide the extent of the interaction scope to capture neighborhood highlight intuitive, overcoming the catch-22 of balancing execution and computational costs. The module diagram is shown in Figure 8.

## 3. Data Collection and Experimental Setup

At present, there is no publicly available image dataset specifically designed for coal gangue on underground belt conveyors, and the underground environment is harsh, with high risk factors and insufficient lighting, which poses a huge challenge to data collection. Therefore, the data used in the experiment come from image samples collected in different scenarios (coal gangue samples are from Taiyuan, Shanxi), including videos taken by a track assessment robot (model ZDX12, manufactured by a company in Taiyuan, Shanxi, China) from a company in Taiyuan, Shanxi Area, China, amid the operation of a transport belt. The robot body is equipped with an intrinsic safety “dual spectrum” pan tilt camera, which uses a 30× optical zoom and 1080P visible light imaging and has functions such as wireless communication, autonomous intrinsic safety charging, and data storage and querying. The belt runs at a speed of 4 m/s. To ensure image clarity, the original resolution of the pan tilt camera is set to 3840 × 2160, and the robot captures frames at a speed of 40 frames/s.

In order to reduce computational costs and improve network performance, Python 3.9 batch processing was used to first adjust the image resolution to 400 × 600 to implement RetinexFormer, and then Roboflow software was used to label and visualize the images. The dataset was stored in YOLO format. It should be noted that in order to prevent image distortion and incompleteness caused by modifying image pixels, the image is first scaled proportionally, and then the boundary fill algorithm is used to supplement the edges of the image. The filling strategy is domain-based filling. In order to enhance the recognition ability of the network model, all images are preprocessed through filtering, horizontal flipping, mirror flipping, brightness adjustment, and other methods.

**Figure 9:**
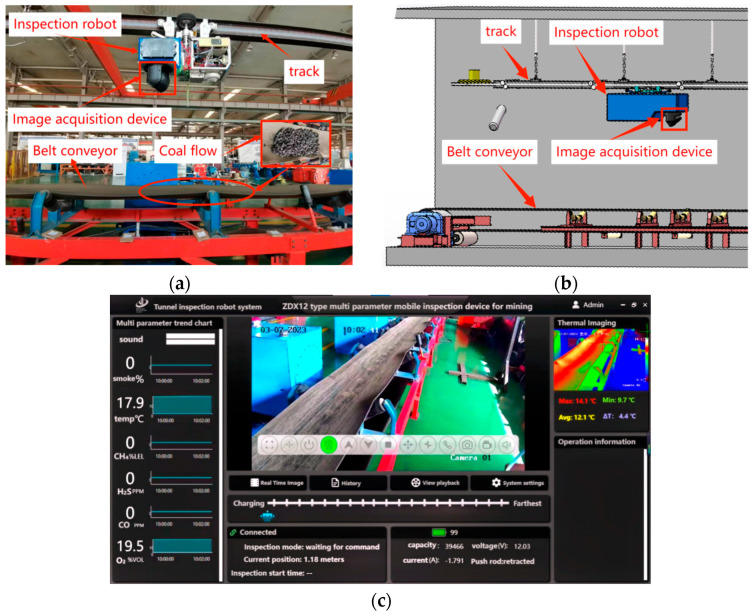
Experimental environment and hardware equipment. (**a**) Map of the experimental site; (**b**) 3D simulation diagram for identifying coal gangue in underground tunnels; (**c**) Monitoring interface.

All image samples are classified into six categories based on different levels of lighting: Normal_light gangue, Normal_light coal, and Normal_light coal gangue mixture; Low_light gangue, Low_light coal, and Low_light coal gangue mixture; and High_light gangue, High_light coal, and High_light coal gangue mixture. Each image contains objects of different sizes, uneven distribution, and varying quantities, with complex features such as accumulation and occlusion. Six datasets are used, each consisting of 1400 images, totaling 8400 image samples and 25,228 data labels. Among them, each dataset was randomly divided into a training set (5880 images), validation set (1680 images), and testing set (840 images) in a ratio of 7:2:1, so that the nine types of images were evenly distributed in the total training set, validation set, and testing set. The detailed dataset samples are shown in Table 1, and the testing environment and hardware facilities are shown in Figure 9 and Table 2.

## 4. Experimental Results

### 4.1. Experimental Environment and Evaluation Indicators

#### 4.1.1. Experimental Environment Configuration

This study is based on YOLOv8 and uses a custom coal gangue dataset to pretrain a model with initialized weights. To make the network adapt to training faster, a learning rate decay strategy is used. Arbitrary Angle Plummet (SGD) is set as the optimizer for parameter upgrades to guarantee superior convergence and generalization capacity of the model, and set the learning rate energy coefficient to 0.9 to avoid the model falling into nearby optima or skipping global optima. Amid the training tests, the complete dataset was iterated 200 times, with a group estimate of 16. The input picture resolution was set to 640 × 640. The introductory learning rate (lr0) is 0.01, and the weight decay coefficient (weight_decay) is 0.0005 to anticipate overfitting. The remaining environmental configurations are shown in Table 3.

#### 4.1.2. Description of Evaluation Indicators

In order to comprehensively and objectively assess the execution of the proposed model, Accuracy, Recall, and Mean Average Precision (mAP) are utilized as pointers to measure and assess the research results.

Particularly, precision Precision is the proportion of accurately anticipated positive training targets by the model to all anticipated positive course targets, and Recall is the number of accurately identified positive tests within the prediction results. When Recall=100%, there are no missed detections; when Precision=100%, no false positives are present. The defined formulas are as follows (1), (2), and (3):

(1) $$\mathrm{Precision}=\frac{TP}{TP+FP}$$

(2) $$\mathrm{Recall}=\frac{TP}{TP+FN}$$

(3) $$IoU=\frac{A\cap B}{A\cup B}$$

Among them, TP (True Positive) speaks to the number of genuine positive tests, that is, the predicted results and actual labels of the test set are both coal; FP (False Positive) alludes to the number of untrue positive tests, where the predicted result of the test set is coal but the actual label is gangue; FN (False Negative) speaks to the number of false negative tests, that is, the predicted result of the test set is gangue, but the actual label is coal; and finally, TN (True Negative) speaks to the number of genuine negative tests, that is, when both the predicted result and the actual label of the test set are gangue. In addition, IoU (Intersection over Union) alludes to the proportion of the crossing point and union between the predicted box A and the actual box B, utilized to measure the Precision of the predicted box. The IoU value of the model is set to 0.7. When the IoU value is more prominent than the set edge of 0.7, a true positive will be decided, whereas a false positive will cause the IoU value to be lower than this limit.

In practice, accuracy and recall are mutually restrictive, and comparing them separately can lead to ambiguity. The AP (Average Exactness) is the range beneath the precision recall curve, speaking to the normal precision of the model for each category at different recall rates. The formula is defined as follows:

(4) $$AP=\int_{0}^{1}PRdr$$

The AP of all classes is obtained, and the normal mAP (mean average precision) of these AP values beneath a pre-specified IoU limit is calculated to comprehensively measure the execution of the model in predicting target position and category. AP is usually one of the most vital markers for assessing the execution of protest detection calculations. The equation is characterized as follows:

(5) $$mAP=\frac{1}{C}\sum_{i=1}^{C}AP_{i}$$

where C speaks to the number of categories within the dataset. The higher the mAP value, the better the execution of the model.

### 4.2. Analysis of Image Preprocessing Results of Retinexformer Module

Due to the interference of dust, stray light, and other factors in the underground environment of coal mines, the images collected from coal flow monitoring can suffer from problems such as uneven lighting, blurred details, and complex noise, resulting in the loss of picture highlight data. In order to obtain more detailed data around the picture, eliminate the impact of environmental lighting components on picture quality and hence influence the recognition accuracy of the model, the Retinexformer module is utilized to preprocess the captured unique picture, alter the brightness and contrast of the picture, make the initial picture clear, retain self-evident edge highlights, and make it simple to recognize. In order to encourage validate the picture handling impact of Retinexformer, a few prevalent picture improvement algorithms, namely SCI [29], Kind++ [30], and Zero DCE [31], were chosen for comparison. The pictures before and after preprocessing are shown in Table 4.

It can be seen that although there are lights illuminating the coal mine underground, the brightness and area of the lights are very limited. The original image has insufficient light intensity and is accompanied by noise. The details in the dark areas are weakened, and information loss is obvious. After being improved by five different calculations, the brightness of the initial picture has been progressed, and the darker ranges have been essentially upgraded. The general brightness and contrast of the picture have been significantly improved, but there are differences in their impacts. The images processed by SCI have serious color distortion problems, especially in areas with high contrast in gangue images, where there is a severe overexposure phenomenon. After processing with KinD++, the image is prone to noise, and it can also amplify the noise in certain areas. The edges of all three images show obvious color distortion issues. Zero-DCE is weaker at enhancing image brightness compared to the other methods, and does not eliminate noise in low-brightness areas of the image. After enhancement, distortion occurs in the image, especially in gangue images, where the texture details on the gangue surface are unclear and there is a certain degree of loss. The general impact of the picture preprocessed by the Retinexformer calculation utilized in this article is obvious, and the brightness and contrast are incredibly improved, noticeably improving the brightness and contrast of low-light regions within the picture. The brighter areas of the image are not overexposed, which to some extent suppresses noise and avoids noise amplification caused by increasing contrast. It improves the common overexposure and color distortion problems in ordinary enhancement methods and preserves edge features and details in extremely dark areas better than other methods.

In addition, to equitably assess the viability of the picture enhancement strategy in this article, the Peak Signal-to-Noise Ratio (PSNR) and Structural Similarity (SSIM) were utilized as objective assessment criteria.

PSNR is an image quality evaluator based on the error sensitivity between corresponding pixel points, which is the ratio of the energy of the peak signal of the image pixel to the average energy of the noise; it can be used to evaluate the degree of distortion of the image, measured in dB. The larger the value, the smaller the distortion and the better the subjective perception of the human eye. The formula is expressed as follows:

(6) $$MSE=\frac{1}{mn}{\sum_{i=0}^{m-1}\sum_{j=0}^{n-1}\left|\left|I\left(i,j\right)-K\left(i,j\right)\right|\right|}^{2}$$

(7) $$\mathrm{PSNR}=10\log_{10}\left(\frac{Max_{Value}^{2}}{MSE}\right)=20\log_{10}\left(\frac{{Max}_{Value}}{\sqrt{MSE}}\right)=10\log_{10}\left(\frac{{\left(2^{p}-1\right)}^{2}}{MSE}\right)$$

where MSE (Mean Square Error) is the mean square error between the corresponding pixel points (i.e., the same coordinate points) of the current image and the reference image; m,n speak to the height and width of the picture, respectively; p is the number of bits per pixel; and $Max_{Value}$ is the maximum value of the color of the image points.

SSIM is based on the assumption that the human eye can extricate organized data from pictures, which is more in line with human visual recognition than conventional strategies. It measures the similarity between the upgraded picture and the reference picture from three perspectives: brightness, contrast, and structure, with a value range of [0, 1]. The bigger the value, the better the image enhancement’s impact and the smaller the distortion. The particular equation is as follows:

(8) $$l(X,Y)=\frac{2\mu_{X}\mu_{Y}+C_{1}}{\mu_{X}^{2}+\mu_{Y}^{2}+C_{1}},c(X,Y)=\frac{2\sigma_{X}\sigma_{Y}+C_{2}}{\sigma_{X}^{2}+\sigma_{Y}^{2}+C_{2}},s(X,Y)=\frac{\sigma_{XY}+C_{3}}{\sigma_{X}\sigma_{Y}+C_{3}}$$

(9) $$SSIM(X,Y)={\left[l(X,Y)\right]}^{\alpha }\cdot {\left[c(X,Y)\right]}^{\beta }\cdot {\left[s(X,Y)\right]}^{\gamma }$$

where l(X,Y) is the brightness of the picture; c(X,Y) is the image contrast; s(X,Y) is the image structure; $\mu_{X},\mu_{Y}$ represent the mean of images X and Y, respectively; $\sigma_{X},\sigma_{Y}$ represent the standard deviation of images X and Y, respectively; $\sigma_{XY}$ represents the covariance of images X and Y; C_1_, C_2_, and C_3_ are constants used to avoid situations where the denominator is 0; and α,β,γ>0 are utilized to alter the extent of the three parts.

**Figure 10:**
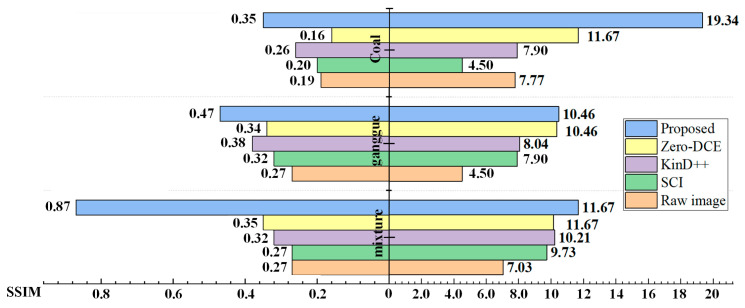
PSNR and SSIM values of preprocessed images.

The objective evaluation results of images under different methods are shown in Figure 10. A high PSNR value indicates that the difference between the reconstructed image and the original image is small and the quality is high. The closer the SSIM value is to 1, the higher the structural similarity and quality between the reconstructed image and the original image. On the left side of the y-axis in Figure 10 are the SSIM data indicators, and on the right side of the y-axis are the PSNR data indicators. The blue bars correspond to the proposed method. From the data distribution, it can be seen that the PSNR and SSIM values of the images processed by the RetinexFormer algorithm have increased by 148% and 222%, respectively, and its SSIM value is closest to 1, showing a significant advantage. This indicates that the distortion degree of the image processed by our method is the smallest and the enhancement effect is the best.

The objective assessment results of images under different strategies are shown in Figure 10. The next PSNR value shows a smaller distinction and higher quality between the remade picture and the initial picture. The closer the SSIM value is to 1, the higher the structural similarity and the greater the quality between the reproduced picture and the initial picture. Clearly, the preprocessed picture is clearer than the initial picture, with improved PSNR and SSIM values. The Retinexformer calculation improved the PSNR and SSIM values of the prepared pictures by 148% and 222%, respectively, and its SSIM value was closest to 1, showing a noteworthy advantage. This indicates that the picture distortion degree caused by this strategy is the smallest and the enhancement effect is the best.

### 4.3. Experimental Results of Training the Proposed Improved Model

**Figure 11:**
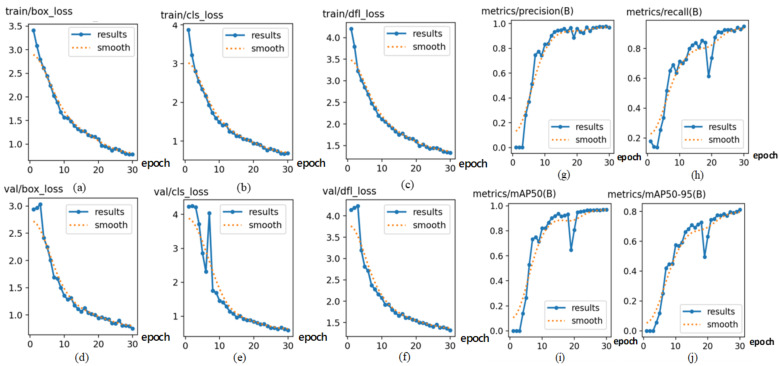
The performance indicators of the proposed improved YOLOv8 model.

The distinctive performance markers of the training and validation sets in this study are shown in Figure 11. The first three columns portray the box loss (Figure 11a,d), classification loss (Figure 11b,e), and question loss (Figure 11c,f) of the improved YOLOv8 model. The three curves within the first three columns demonstrate the drift of loss, where the x-axis refers to the time on the training set in cycles and the y-axis to the general loss value. From the curve, it can be seen that as the training continues, the general loss value persistently diminishes and inevitably stabilizes. The results demonstrate that the improved YOLOv8 model has great fitting performance and high steadiness and precision. The final two columns speak to the PR curves (Figure 11g–j), where the x-axis refers to the training time in cycles and the y-axis refers to precision and recall. These curves illustrate the assessment of question detection performance as the certainty limit changes: the closer the curve value is to 1, the higher the accuracy of the model. From Figure 11, it can be seen that the improved YOLOv8 show is successful.

**Figure 12:**
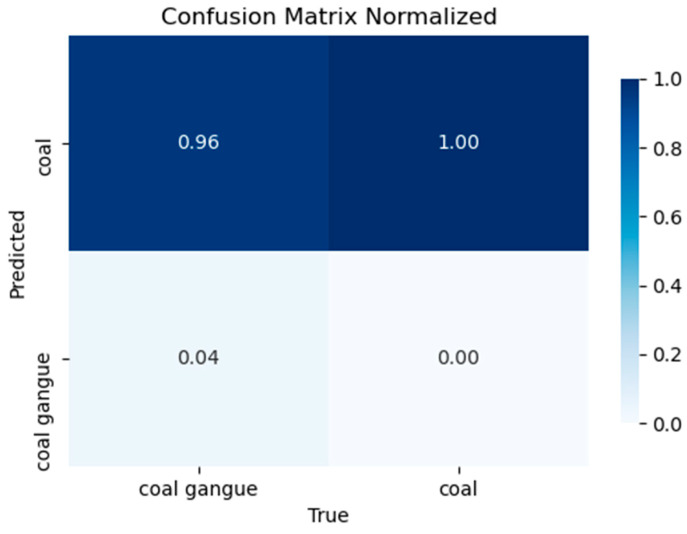
The confusion matrix of the proposed improved YOLOv8 model.

Figure 12 shows the confusion matrix of the proposed improved YOLOv8 model, describes its prediction accuracy of middling coal gangue mixed into the coal pile in the data set, and explains the relationship between the predicted value and the true value. The rows represent the true label, the columns show the predicted category, and the diagonal element shows the correct detection rate. A value of 0 corresponds to white, indicating that the model detects coal gangue as the sample, but the detection rate of a true coal block is 0. The plate directly above 0 indicates that the model detects coal blocks as samples, and its actual detection rate for coal blocks is 100%, reflecting the extremely low false detection rate and high accuracy of the network.

**Figure 13:**
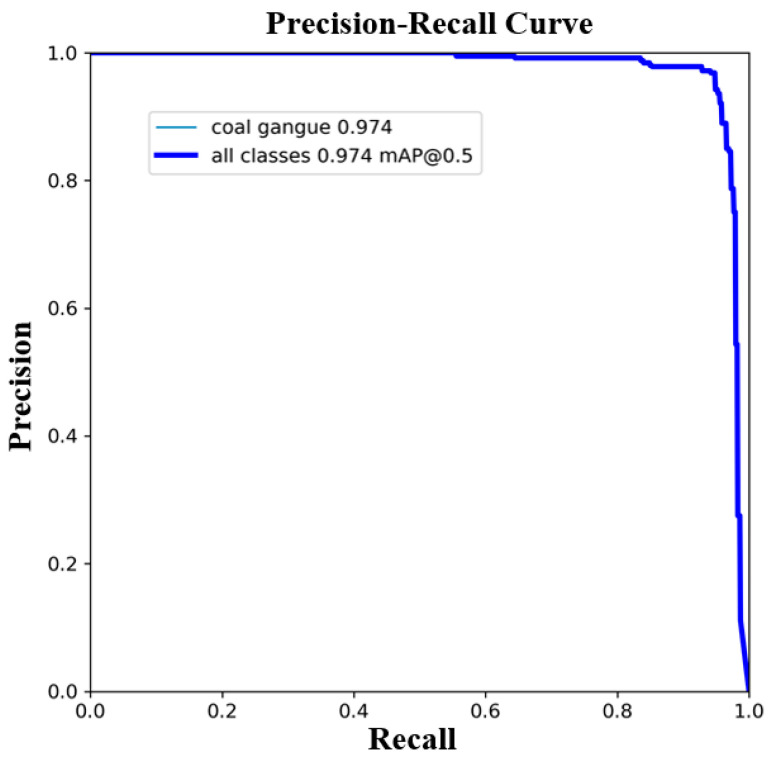
Improved YOLOv8 model’s PR curve.

Figure 13 shows the PR curve of the model. It can be seen that the rate of change in precision increases with the increase in review rate. The PR curve of the proposed model is near to the upper right corner, demonstrating that the improved model can maintain high precision at lower certainty limits. The AUC (area under the PR curve) is moderately expansive, demonstrating that the model includes a high recall rate while maintaining high precision, indicating that the general performance of the model is good under different certainty edges. In addition, the PR curve is additionally exceptionally smooth, showing that the proposed model produces small disruptions or vacillations at different certainty edges, showing a moderately steady relationship between recall and precision.

To illustrate the higher exactness of the model proposed in this study, a few images were chosen from the test dataset. Table 5 presents the coal gangue location results of the proposed improved YOLOv8 show and the initial YOLOv8 model. From the first column in the graph, it can be seen that the initial YOLOv8 model has instances of FN and FP predictions. For example, under low- and high-light conditions, the original YOLOv8 model mistakenly identified coal gangue as coal blocks without labeling them; under normal lighting conditions, the original YOLOv8 model ignored certain coal gangue objects and mistakenly identified some coal blocks as coal gangue and labeled them. However, these issues did not show up within the recognition results of the proposed improved YOLOv8 model. In addition, the accuracy level of the proposed improved model for recognizing gangue is altogether higher than that of the initial model. The test results show that the improved YOLOv8 model essentially meets the requirements of coal gangue recognition tasks, with low false detection and missed detection rates, and can accomplish accurate detection, which has more viable application value.

In summary, the proposed improved YOLOv8 model has great fitting performance and high reliability, precision, and accuracy. From the PR curve, it can be seen that the model performs well, with high recall and precision. From the results, it can be seen that the location precision is high and the detection time is short. For a few coal and gangue pictures that cannot be rapidly and precisely judged by people, the target can be precisely recognized, and coal and gangue can be precisely distinguished and found in each picture. Subsequently, the improved YOLOv8 model plays a vital role in the division of coal and gangue.

### 4.4. Analysis of Ablation Experiment Results

Compared to the original network that performs detection tasks separately, adding different modules to the backbone feature network may affect the detection results to some extent. This section confirms the viability of each proposed advancement module on location tasks through removal tests, and assesses their effect on performance by specifically expelling these enhancement modules. The test utilized the same dataset, computer program, and equipment hardware, and the results are shown in Table 6.

Within the table above, the first row of information shows the detection results of the first YOLOv8 model for low-light pictures, which can be taken as the pattern for this test. The second to fourth lines show the test results after including as it were one module, individually clarifying the effect of the low-light improvement module, highlight extraction module, lightweight module, and attention component module on the performance of the whole model. Watching mAP@0.5 and mAP@0.5:0.95 has expanded by up to 7.48% and 32.30% Compared to the initial YOLOv8, separately. It is worth noting that after adding the BiFPN module separately, the model’s mAP@0.5 and mAP@0.5:0.95 decreased slightly. This is because the strong feature extraction ability of the BiFPN module leads to overfitting in the model, which also reflects the importance of incorporating the MCA module, as shown in line 10. The 6th to 15th lines show the arrangement and combination of different modules added, and these improvements have resulted in an average increase of 7.533% and 25.547% in the mAP@0.5 and mAP@0.5:95 of the model. The last line shows the final experimental results of the model. The mAP@0.5 and mAP@0.5:95 of the model have improved by 10.49% and 36.62% compared to the original model. In addition, the original YOLOv8 network has a computational complexity of 105.47 GFLOPs and a computation time of 2.133 h, while the improved network has a computational complexity of 8.1 GFLOPs and a computation time of 1.701 h. This proves that the proposed improved YOLOv8 model has an enhanced feature extraction capability, accuracy, and recall rate, while also ensuring detection speed and meeting real-time requirements.

### 4.5. Comparative Experiment

#### 4.5.1. Comparative Analysis of Different Attention Mechanisms

In order to further explore the effectiveness of the MCA attention mechanism proposed in this study at improving the YOLOv8 model, four sets of comparative tests were designed. Four popular attention modules—including efficient multi-scale attention (EMA), coordinate attention mechanism (CA), pyramid split attention (PSA), and multi-head self-attention (MHSA)—are used to improve the YOLOv8 model in the same way, and the corresponding models are trained and tested on the training and testing sets. The experimental results are shown in Table 7.

The EMA module divides the channel dimension into multiple sub-features to evenly distribute spatial semantic features within each feature group, but it focuses more on the information of the current window or local area and it is difficult to effectively integrate global information. The CA module decomposes channel attention into two 1D feature encoding processes that aggregate features along different directions, forming feature maps that are sensitive to direction and position. However, it mainly focuses on attention in the channel dimension and is not flexible enough for spatial attention requirements in specific scenarios such as underground. The PSA module preserves the potential loss of high-resolution information in the original deep convolutional neural network through downsampling, while the MHSA module models the dependency relationships at different positions in the input sequence. However, they have high computational complexity and memory consumption, and also require high hardware resources.

The analysis of experimental results shows that the YOLOv8 model improved by the MCA module proposed in this study simultaneously improves attention in the channel, height, and width dimensions, with almost no additional overhead, exhibiting the highest accuracy, recall, and mAP, which are 96.4, 96.7, and 86%, respectively. The performance is better than all the compared attention modules, which indicates that the MCA attention module proposed in this study can effectively improve the detection performance of middling coal and gangue in underground low-light environments.

#### 4.5.2. Comparative Analysis of Different SOTA (State of the Art) Models

**Figure 14:**
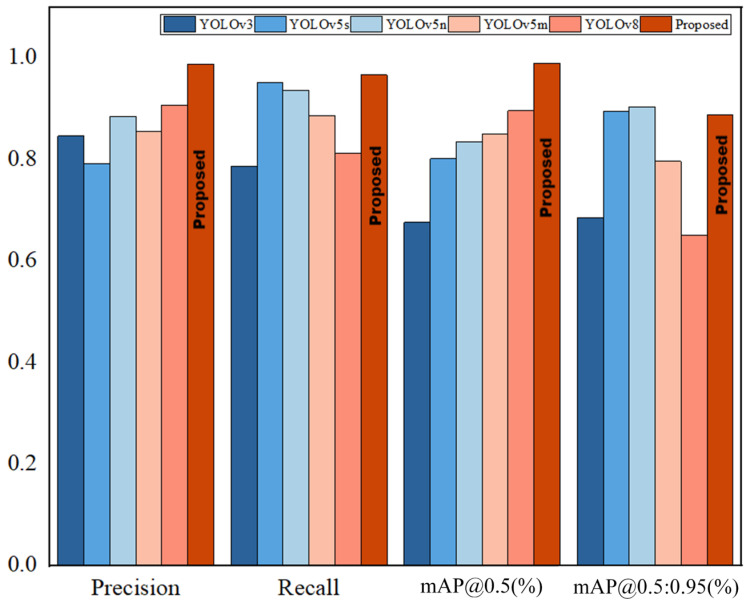
Comparison results of different SOTA models.

In order to confirm the viability of the calculation proposed in this paper, distinctive single-stage acknowledgment calculations such as YOLOv3 [32] and YOLOv5 networks were tested on the coal gangue dataset, and the test results were compared to assess their performance. In order to guarantee the validity of the test, the same exploratory conditions were utilized, with the first YOLOv8 model as the standard. The performance comparison results of the different algorithms are shown in Figure 14.

As shown in the table above, the proposed improved YOLOv8 model has mAP@0.5 and mAP@0.5:0.95 values 46.44% and 29.63% higher than YOLOv3, 16.47% and −0.78% higher than YOLOv5s, 23.59% and −1.70% higher than YOLOv5n, and 18.56% and 11.55% higher than YOLOv5m. The test results show that the model proposed in this paper has solid performance.

#### 4.5.3. Comparative Analysis of Results from Different Datasets

The generalization capacity of a model alludes to its capacity to adapt to new information. In order to confirm the generalization capacity of the proposed model, this paper supplanted distinctive datasets with comparative highlights, such as dense scenes, multi-scale objects, and potentially occluded instances, for comparative experiments, such as the public datasets CrowdHuman [33] and COCO, rock datasets, parts datasets, conveyor belt foreign object datasets, etc. The same test conditions were utilized as mentioned above, with the improved YOLOv8 model as the standard. The results from the distinctive datasets are shown in Table 8.

From these results, it can be seen that the improved YOLOv8 model in this study accomplished great results on diverse datasets. CrowdHuman belongs to a typical dense, multi-scale, and mutually occluded object detection scene. The proposed improved model achieves an average accuracy, recall, and mAP@0.5 (%) value of 0.871, 0.873, and 0.919 in three categories. Six different types of target images, including umbrellas, were selected from the COCO dataset for recognition, with average accuracy, recall, and mAP@0.5 (%) values reaching 0.915, 0.959, and 0.975. The proposed improved model is applicable to rock datasets with an average mAP@0.5 (%) of 0.941. The average mAP@0.5 (%) value in the parts dataset reached 0.916, and the average mAP@0.5 (%) value in the foreign object dataset reached 0.800. This indicates that the proposed improved model is effectively applied to the recognition of foreign objects of different scales. The above investigation demonstrates that the improved model in this study has solid generalization capacity.

## 5. Discussion and Future Outlook

### 5.1. Discussion

Machine vision object detection algorithms in practical engineering applications often fail to balance both target recognition accuracy and system execution speed. In order to meet the demands of machine vision for genuine independent recognition and high-precision intelligent classification in complex situations within the coal industry, a new “hardware friendly” coal gangue image recognition algorithm is proposed, using YOLOv8 as the backbone network, the Retinexformer module for dim light image preprocessing, and introducing a repeated weighted bi-directional feature extraction module (BiFPN), a lightweight module (RepGhost), and a multi-dimensional attention mechanism (MCA) to further improve model accuracy and performance.

From the research, it can be seen that each proposed module successfully makes strides the detection accuracy and speed of the calculation: the testing accuracy of the proposed improved model is as tall as 0.988, mAP@0.5 (%) value and mAP@0.5.95 (%) value is 10.49% and 36.62% higher than the original YOLOv8 model, respectively; Compared to YOLOv3, it has increased by 46.44% and 29.63% respectively, and has increased by an average of 19.54% and 3.02% compared to the YOLOv5 series; The inference speed reached 8.1 GFLOPS, which is 92.32% higher than the original YOLOv8 model; The detection experiment results on different datasets are good, indicating that the model can digest new data and make accurate predictions, with excellent generalization ability.

In spite of the fact that the sorts of coal and gangue change completely different locales, with marginally distinctive compositions and appearances, the strategy proposed in this article is basic in hardware, environmentally friendly, and has strong flexibility and universality. It does not require large-scale transformation of coal flow lines and can be applied to surface coal preparation plants, direct sorting of underground raw coal, or transplantation to other coal mines through parameter adjustment to solve production problems.

### 5.2. Future Outlook

In future work, we will open and encourage expansion and development of the dataset proposed in this paper. For example, we will analyze the effects of natural changes such as mine humidity and temperature on the characteristics of coal gangue, and attempt to differentiate “average coal” (coal gangue blend, whose surface has both the characteristics of coal and gangue) from the coal stream, so that the model has superior precision and robustness, enhance its application potential in industrial practice.

## Figures and Tables

**Table 1:** Partial sample diagram of coal, coal gangue mixture, and gangue.

Light Intensity	Coal	Coal Gangue Mixture	Gangue
Low_light	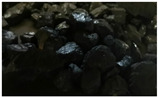	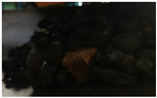	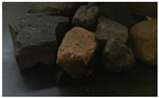
Normal_light	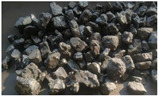	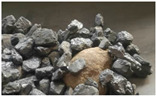	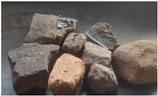

**Table 2:** Hardware facility parameters.

Facility Name	Technical Parameter Settings
Camera capture frame rate	50 FPS
Shooting resolution	3840 × 2160
Robot endurance	>20 h
Belt speed	4 m/s
Bandwidth	1400 mm
Other performance	Dust-proof, explosion-proof, rust-proof

**Table 3:** Environmental configuration.

Configuration Name	Configuration Parameter
Operating system	Windows 10
CPU	Intel(R) Core (TM) i5-7500 CPU @ 3.40 GHz 3.40 GHz
GPU	NVIDIA GeForce RTX 4060 Ti
Python	3.9
PyTorch	1.11.0
Cuda	11.3.1

**Table 4:** Comparison of image preprocessing effects using different low-light enhancement algorithms.

Algorithm	Coal	Coal Gangue Mixture	Gangue
NONE	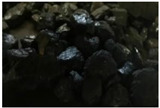	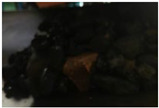	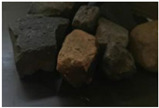
SCI	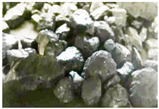	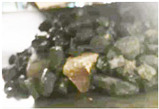	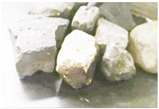
KinD++	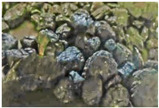	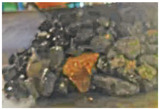	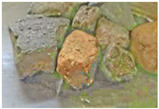
Zero-DCE	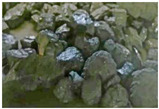	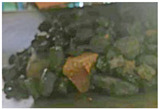	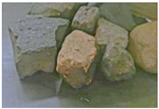
Proposed	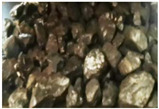	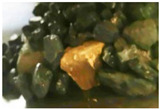	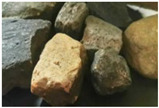
NONE	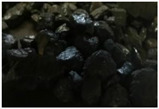	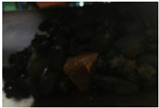	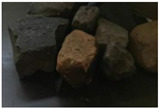

**Table 5:** Partial results of coal gangue detection before and after model improvement.

	Original YOLOv8 Model	Proposed
Dark_light	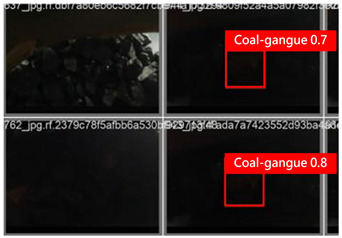	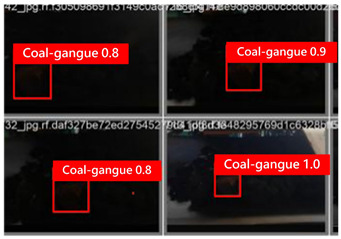
High_light	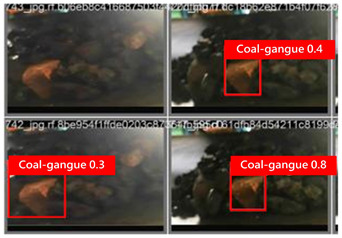	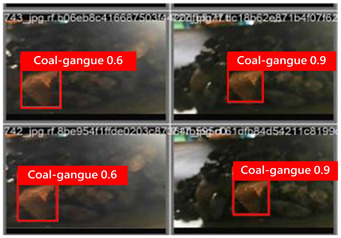
Normal_light	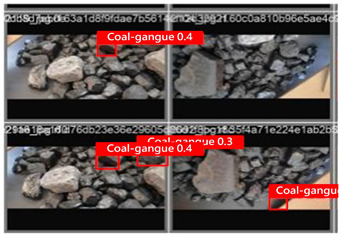	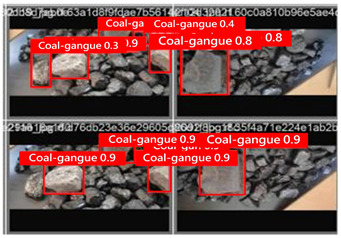

**Table 6:** Experimental data on different combinations of improved YOLOv8 modules.

	Retinexformer	BiFPN	RepGhost	MCA	Precision (×10^−1^)	Recall (×10^−1^)	mAP@0.5(%) (×10^−1^)	mAP@0.5: 0.95 (%) (×10^−1^)	FLOPs (G)
1					9.07	8.12	8.96	6.50	105.47
2	√				9.31	8.40	9.27 (+3.46%)	7.16 (+10.15%)	71.62
3		√			9.47	8.30	9.22 (+2.90%)	5.96 (−8.31%)	55.88
4			√		9.68	9.76	9.54 (+6.47%)	8.58 (+32.00%)	51.82
5				√	9.64	9.67	9.63 (+7.48%)	8.60 (+32.30%)	54.63
6	√	√			9.31	8.40	9.27 (+3.46%)	7.16 (+10.15%)	20.92
7	√		√		9.69	9.67	9.50 (+6.02%)	8.66 (+33.23%)	25.66
8	√			√	9.69	9.66	9.56 (+6.70%)	8.66 (+33.23%)	19.81
9		√	√		9.70	9.63	9.64 (+7.59%)	8.60 (+32.31%)	26.74
10		√		√	9.72	9.75	9.60 (+7.14%)	7.60 (+16.92%)	20.75
11			√	√	9.76	9.42	9.65 (+7.70%)	7.62 (+17.23%)	23.22
12	√	√	√		9.75	9.63	9.64 (+7.59%)	8.12 (+24.92%)	16.77
13	√	√		√	9.78	9.86	9.94 (+10.93%)	8.68 (+33.54%)	14.80
14	√		√	√	9.79	9.57	9.81 (+9.49%)	8.79 (+35.23%)	14.82
15		√	√	√	9.85	9.51	9.74 (+8.71%)	7.71 (+18.62%)	15.00
16	√	√	√	√	9.88	9.66	9.90 (+10.49%)	8.88 (+36.62%)	8.1

**Table 7:** Experimental data of YOLOv8 combinations with different attention mechanisms.

	Algorithms	Precision (×10^−1^)	Recall (×10^−1^)	mAP@0.5 (%) (×10^−1^)	mAP@0.5:0.95 (%) (×10^−1^)
1	YOLOv8	9.07	8.12	8.96	6.50
2	YOLOv8 + MCA	9.64	9.67	9.63 (+7.48%)	8.60 (+32.30%)
3	YOLOv8 + EMA	8.92	9.76	8.85 (−1.24%)	4.76 (−26.77%)
4	YOLOv8 + CA	7.92	8.48	7.46 (−16.74%)	5.26 (−19.07%)
5	YOLOv8 + PSA	7.48	7.12	7.64 (−14.73%)	5.31 (−18.31%)
6	YOLOv8 + MHSA	8.06	8.07	8.64 (−3.57%)	5.67 (−12.77%)

**Table 8:** Test results for different datasets.

CrowdHuman	COCO
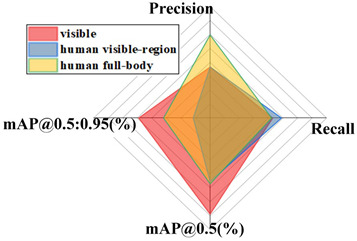	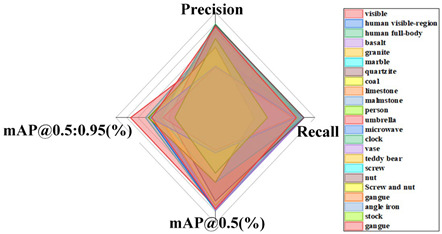
Rock Dataset	Part Dataset
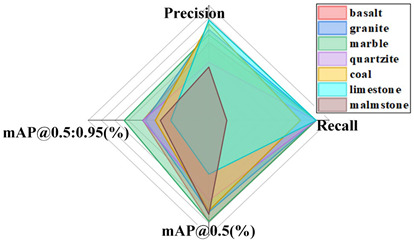	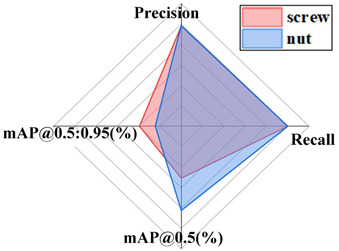
Foreign object Dataset	
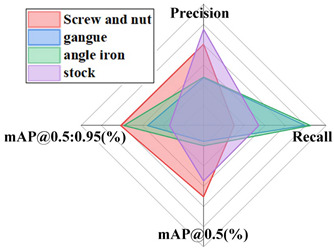	

## Data Availability

The data presented in this study are available on request from the corresponding author. The data are not publicly available due to privacy reasons.

## References

[1] Zhou W., Wang H., Wang L., Feng A., Li L., Zhu J. (2024). Research on separation mechanism of coal gangue photoelectric sorting recognition. Physicochem. Probl. Miner. Process..

[2] Xun Q., Yang Y., Liu Y. (2023). Research on the Strong Generalization of Coal Gangue Recognition Technology Based on the Image and Convolutional Neural Network under Complex Conditions. ACS Omega.

[3] Shi F., Li X., Cao Y., Bai B. (2023). The Feasibility Analysis of “Ecological Photovoltaics” from Coal Gangue Mountains. Sustainability.

[4] Hao Y., Guo X., Yao X., Han R., Li L., Zhang M. (2022). Using Chinese Coal Gangue as an Ecological Aggregate and Its Modification: A Review. Materials.

[5] Xue B., Zhang Y., Li J., Wang Y. (2022). A review of coal gangue identification research-application to China′s top coal release process. Environ. Sci. Pollut. Res. Int..

[6] Li N., Gong X. (2021). An Image Preprocessing Model of Coal and Gangue in High Dust and Low Light Conditions Based on the Joint Enhancement Algorithm. Comput. Intell. Neurosci..

[7] Zhao Y., Wang S., Cheng G., He L. (2022). Study on coal and gangue recognition method based on the combination of X-ray transmission and diffraction principle. Energy Sources, Part A: Recovery, Utilization, and Environmental. Effects.

[8] Lai W., Zhou M., Hu F., Bian K., Song H. (2020). A study of Multispectral Technology and Two-dimension Autoencoder for Coal and Gangue Recognition. IEEE Access.

[9] Wang W., Lv Z., Lu H. (2018). Research on methods to differentiate coal and gangue using image processing and a support vector machine. Int. J. Coal Prep. Util..

[10] Cao X., Wei H., Wang P., Zhang C., Huang S., Li H. (2022). High Quality Coal Foreign Object Image Generation Method Based on StyleGAN-DSAD. Sensors.

[11] Wang X., Guo Y., Wang S., Cheng G., Wang X., He L. (2022). Rapid detection of incomplete coal and gangue based on improved PSPNet. Measurement.

[12] Wang L., Wang X., Li B. (2023). Data-driven model SSD-BSP for multi-target coal-gangue detection. Measurement.

[13] Lai W., Hu F., Kong X., Yan P., Bian K., Dai X. (2022). The study of coal gangue segmentation for location and shape predicts based on multispectral and improved Mask R-CNN. Powder Technol..

[14] Zhang Y., Wang J., Yu Z., Zhao S., Bei G. (2022). Research on intelligent detection of coal gangue based on deep learning. Measurement.

[15] Yang Y., Li D., Guo Y., Wang S., Zhao D., Chen W., Zhang H. (2024). Research on coal gangue recognition method based on XBS-YOLOv5s. Meas. Sci. Technol..

[16] Zeng Q., Zhou G., Wan L., Wang L., Xuan G., Shao Y. (2024). Detection of Coal and Gangue Based on Improved YOLOv8. Sensors.

[17] Xue G., Li S., Hou P., Gao S., Tan R. (2023). Research on lightweight Yolo coal gangue detection algorithm based on resnet18 backbone feature network. Internet Things.

[18] Liu Q., Li J., Li Y., Gao M. (2021). Recognition Methods for Coal and Coal Gangue Based on Deep Learning. IEEE Access.

[19] Yan P., Sun Q., Yin N., Hua L., Shang S., Zhang C. (2022). Detection of coal and gangue based on improved YOLOv5.1 which embedded scSE module. Measurement.

[20] Gui F., Yu S., Zhang H., Zhu H. Coal Gangue Recognition Algorithm Based on Improved YOLOv5. Proceedings of the 2021 IEEE 2nd International Conference on Information Technology, Big Data and Artificial Intelligence (ICIBA).

[21] Terven J., Córdova-Esparza D.M., Romero-González J.A. (2023). A Comprehensive Review of YOLO Architectures in Computer Vision: From YOLOv1 to YOLOv8 and YOLO-NAS. Mach. Learn. Knowl. Extraction..

[22] Cai Y., Bian H., Lin J., Wang H., Timofte R., Zhang Y. Retinexformer: One-stage Retinex-based Transformer for Low-light Image Enhancement. Proceedings of the 2023 IEEE/CVF International Conference on Computer Vision (ICCV).

[23] Han K., Wang Y., Tian Q., Guo J., Xu C., Xu C. GhostNet: More features from cheap operations. Proceedings of the IEEE Computer Society Conference on Computer Vision and Pattern Recognition.

[24] Tan M., Pang R., Le Q.V. EfficientDet: Scalable and efficient object detection. Proceedings of the IEEE Computer Society Conference on Computer Vision and Pattern Recognition.

[25] Lin T.Y., Dollár P., Girshick R., He K., Hariharan B., Belongie S. Feature pyramid networks for object detection. Proceedings of the IEEE Conference on Computer Vision and Pattern Recognition.

[26] Liu S., Qi L., Qin H., Shi J., Jia J. Path Aggregation Network for Instance Segmentation. Proceedings of the 2018, IEEE/CVF Conference on Computer Vision and Pattern Recognition (CVPR).

[27] Ghiasi G., Lin T.Y., Le Q.V. NAS-FPN: Learning Scalable Feature Pyramid Architecture for Object Detection. Proceedings of the IEEE/CVF Conference on Computer Vision and Pattern Recognition.

[28] Yu Y., Zhang Y., Cheng Z., Song Z., Tang C. (2023). MCA: Multidimensional collaborative attention in deep convolutional neural networks for image recognition. Eng. Appl. Artif. Intell..

[29] Ma L., Ma T., Liu R., Fan X., Luo Z. Toward Fast, Flexible, and Robust Low-Light Image Enhancement. Proceedings of the 2022 IEEE/CVF Conference on Computer Vision and Pattern Recognition (CVPR).

[30] Zhang Y., Guo X., Ma J., Liu W., Zhang J. (2021). Beyond Brightening Low-light Images. Int. J. Comput. Vision.

[31] Guo C., Li C., Guo J., Loy C.C., Hou J., Kwong S., Cong R. Zero-reference deep curve estimation for low-light image enhancement. Proceedings of the IEEE Computer Society Conference on Computer Vision and Pattern Recognition.

[32] Redmon J. (2018). YOLOv3: An Incremental Improvement. arXiv.

[33] Shao S., Zhao Z., Li B., Xiao T., Yu G., Zhang X., Sun J. (2018). CrowdHuman: A Benchmark for Detecting Human in a Crowd. arXiv.

